# Changes in Tacrolimus and Everolimus Trough Concentrations and Dose Requirements After Initiation and Discontinuation of Letermovir Prophylaxis in Kidney Transplant Recipients: A Single-Center Retrospective Study

**DOI:** 10.3390/jcm15207875

**Published:** 2026-10-13

**Authors:** Junji Uchida, Shunji Nishide, Yuji Takeyama, Taiyo Otoshi, Minoru Kato, Takeshi Yamasaki, Tomoaki Iwai, Toshihide Naganuma, Katsuyuki Kuratsukuri

**Affiliations:** Department of Urology, Graduate School of Medicine, Osaka Metropolitan University, 1-4-3 Asahi-machi, Abeno-ku, Osaka 545-8585, Japan; nishide@omu.ac.jp (S.N.); yuji101@outlook.jp (Y.T.); sun.otoshi@gmail.com (T.O.); l21825e@omu.ac.jp (M.K.); tysy-y@mx5.canvas.ne.jp (T.Y.); iwai@omu.ac.jp (T.I.); spxd48k9@aria.ocn.ne.jp (T.N.); kuratsukuri@omu.ac.jp (K.K.)

**Keywords:** letermovir, cytomegalovirus, kidney transplantation, tacrolimus, everolimus, drug–drug interaction, therapeutic drug monitoring, mTOR inhibitor

## Abstract

**Background/Objectives:** Letermovir is effective for cytomegalovirus (CMV) prophylaxis after kidney transplantation, but cytochrome P450 3A inhibition may alter tacrolimus and everolimus exposure. We evaluated changes in trough concentrations and dose requirements during letermovir initiation and discontinuation in de novo kidney transplant recipients. **Methods:** This single-center retrospective observational study included 20 living-donor kidney transplant recipients with D+/R− or R+ CMV serostatus receiving everolimus-based reduced-tacrolimus immunosuppression and letermovir prophylaxis. Trough concentrations, daily doses, kidney allograft function, CMV outcomes, and safety were assessed descriptively. **Results:** After letermovir initiation, mean tacrolimus trough concentrations increased by approximately 40–60% and everolimus trough concentrations by approximately 35–50%, requiring dose reductions of approximately 45% and 30–40%, respectively. By day 7 after discontinuation, mean tacrolimus and everolimus trough concentrations decreased to 57.4% and 65.9% of baseline, respectively, while daily doses increased to 136.6% and 132.5%, respectively. Group mean serum creatinine and eGFR remained within approximately 10% of baseline. No CMV DNAemia or disease, leukopenia, neutropenia, acute T cell-mediated rejection, or antibody-mediated rejection occurred during letermovir prophylaxis; gastrointestinal symptoms occurred in three recipients. **Conclusions:** Letermovir was associated with rapid, reversible changes in tacrolimus and everolimus trough concentrations and dose requirements. Intensive therapeutic drug monitoring is warranted at both initiation and discontinuation.

## 1. Introduction

Cytomegalovirus (CMV) remains one of the most clinically important opportunistic pathogens after kidney transplantation. CMV infection and disease can cause direct manifestations, including CMV syndrome and tissue-invasive disease, and are associated with indirect consequences such as secondary infections, acute rejection, impaired allograft function, graft loss, and mortality [1]. The risk is highest in CMV-seronegative recipients of kidneys from seropositive donors (D+/R−), although seropositive recipients (R+) remain susceptible to reactivation or reinfection, particularly under intensified immunosuppression [1]. Current international consensus guidance therefore supports risk-adapted CMV prevention using antiviral prophylaxis or preemptive therapy according to donor/recipient serostatus, immunological risk, and institutional practice [1].

Valganciclovir has traditionally been used for CMV prophylaxis in high-risk kidney transplant recipients, but its use is complicated by myelotoxicity and renal function-dependent dose adjustment during the early post-transplant period [1,2]. In a pivotal randomized phase 3 trial involving D+/R− kidney transplant recipients, letermovir administered for up to 200 days was noninferior to valganciclovir for preventing CMV disease through 52 weeks and was associated with substantially less leukopenia or neutropenia [2]. A subsequent post hoc analysis highlighted the practical advantage of fixed daily letermovir dosing across kidney-function strata, whereas valganciclovir frequently required renal function-based dose modification [3].

Everolimus-based immunosuppression may also influence CMV risk. Randomized kidney-transplant studies have reported lower CMV infection or treatment rates with everolimus plus reduced calcineurin inhibitor exposure than with mycophenolate-based regimens [4,5]. However, these studies were not designed to evaluate universal letermovir prophylaxis, and the clinical implications of combining letermovir with everolimus-based reduced-tacrolimus immunosuppression remain incompletely characterized.

Although letermovir has a favorable hematologic safety profile, clinically important drug–drug interactions remain a concern. Letermovir inhibits CYP3A and can increase exposure to immunosuppressants with narrow therapeutic indices [6]. In healthy volunteers, letermovir increased tacrolimus area under the concentration–time curve (AUC) approximately 2.4-fold [7]. For everolimus, a physiologically based pharmacokinetic model predicted an approximately 2.5-fold increase in AUC during letermovir coadministration [8]. These data support close therapeutic drug monitoring when letermovir is initiated and discontinued [6,7,8]. A recent multicenter real-world kidney-transplant series also documented clinically relevant interactions with tacrolimus and everolimus, but letermovir indications and timing were heterogeneous and only four recipients received everolimus [9].

Accordingly, we conducted a single-center retrospective study to characterize early changes in tacrolimus and everolimus trough concentrations and dose requirements following both initiation and discontinuation of letermovir. We additionally evaluated kidney allograft function, CMV outcomes, rejection, and adverse events in de novo kidney transplant recipients receiving letermovir prophylaxis with everolimus-based reduced-tacrolimus immunosuppression.

## 2. Materials and Methods

### 2.1. Study Design and Patients

This was a single-center, retrospective observational study of de novo kidney transplant recipients who underwent living-donor kidney transplantation at the Department of Urology, Osaka Metropolitan University Hospital. Patients who underwent living-donor kidney transplantation between June 2024 and November 2025 were screened. Eligible recipients were either CMV donor-seropositive/recipient-seronegative (D+/R−), representing the high-risk group, or recipient-seropositive (R+), representing the intermediate-risk group, and were receiving concomitant tacrolimus and everolimus at the initiation of letermovir prophylaxis. During the study period, 24 patients underwent living-donor kidney transplantation. Four were excluded because they were receiving cyclosporine or mycophenolate mofetil (MMF), rather than concomitant tacrolimus and everolimus, at the initiation of letermovir prophylaxis. The remaining 20 recipients met all eligibility criteria and were included in the final analysis. Recipients were required to have follow-up through approximately 200 days after transplantation with clinical and therapeutic drug monitoring data available for the early post-letermovir discontinuation assessment. No additional exclusion criteria were applied. This study is reported in accordance with the Strengthening the Reporting of Observational Studies in Epidemiology (STROBE) statement [10].

Clinical data were retrospectively obtained from medical records, including recipient and donor characteristics, donor–recipient relationship, ABO compatibility, CMV serostatus, induction/desensitization therapy, maintenance immunosuppression, letermovir exposure, tacrolimus and everolimus trough concentrations and daily doses, kidney allograft function, CMV DNAemia, rejection episodes, and adverse events. To reduce selection bias, all consecutive recipients meeting the eligibility criteria were included. Concomitant medication changes known to affect tacrolimus or everolimus trough concentrations were reviewed during the principal 7-day assessment periods following letermovir initiation and discontinuation.

### 2.2. Immunosuppressive Regimen and Letermovir Prophylaxis

Maintenance immunosuppression consisted of reduced-exposure tacrolimus in combination with everolimus and corticosteroids. Basiliximab was administered as induction therapy according to the institutional protocol. Rituximab was administered at a dose of 150 mg/m^2^ 2 weeks before transplantation when clinically indicated, in accordance with the institutional desensitization protocol for ABO-incompatible kidney transplantation. Tacrolimus and everolimus were generally initiated 3 days before transplantation. The target tacrolimus trough concentrations were 4–7 ng/mL during the first 2 months after transplantation, 3–6 ng/mL from 2 to 6 months, and 2–4 ng/mL thereafter. The target everolimus trough concentration was 3–8 ng/mL. During each 7-day assessment window after letermovir initiation or discontinuation, the tacrolimus target range applicable at baseline was kept unchanged. In particular, when letermovir was discontinued after the 6-month post-transplant transition, the lower tacrolimus target of 2–4 ng/mL had already been adopted before the pre-discontinuation baseline measurement and remained unchanged throughout the subsequent 7-day assessment. The everolimus target range (3–8 ng/mL) was unchanged throughout both assessment windows. No protocol-mandated taper or scheduled change in immunosuppressant target concentrations occurred within either 7-day window.

Letermovir was generally initiated approximately 14 days after kidney transplantation, after stabilization of kidney allograft function and adjustment of immunosuppressant trough concentrations. Letermovir was administered orally at 480 mg once daily, with prophylaxis planned to continue until approximately day 200 after transplantation. Immunosuppressant doses were individually adjusted according to therapeutic drug monitoring. In patients requiring discontinuation of tacrolimus and concomitant cyclosporine therapy, the letermovir dose was reduced from 480 mg/day to 240 mg/day. One recipient required temporary interruption of letermovir because of diarrhea and dehydration and was subsequently converted from tacrolimus to cyclosporine A before letermovir was resumed at 240 mg/day. This recipient contributed to analyses after letermovir initiation while receiving tacrolimus and everolimus. After conversion to cyclosporine, the recipient was not included in the tacrolimus discontinuation analyses. Available everolimus and kidney-function measurements were retained in the primary descriptive analyses; sensitivity analyses excluding this recipient were also performed for the post-discontinuation everolimus assessments.

### 2.3. Therapeutic Drug Monitoring and CMV Surveillance

Tacrolimus and everolimus trough concentrations, daily doses, serum creatinine, and estimated glomerular filtration rate (eGFR) were monitored daily for 7 days after letermovir initiation during hospitalization whenever feasible. Following letermovir discontinuation, these parameters were evaluated immediately before discontinuation (day 0), on days 2 or 3, and on day 7 whenever available. Tacrolimus and everolimus trough concentrations were measured in-house in whole-blood samples using electrochemiluminescence immunoassays (ECLIA) on a cobas e 801 analytical unit of the cobas pro integrated solutions platform (Roche Diagnostics, Mannheim, Germany). Tacrolimus and everolimus concentrations were determined using the Elecsys Tacrolimus and Elecsys Everolimus assays, respectively (Roche Diagnostics GmbH, Mannheim, Germany). eGFR was calculated using the Japanese equation reported by Matsuo et al. [11].

**Figure 1:**
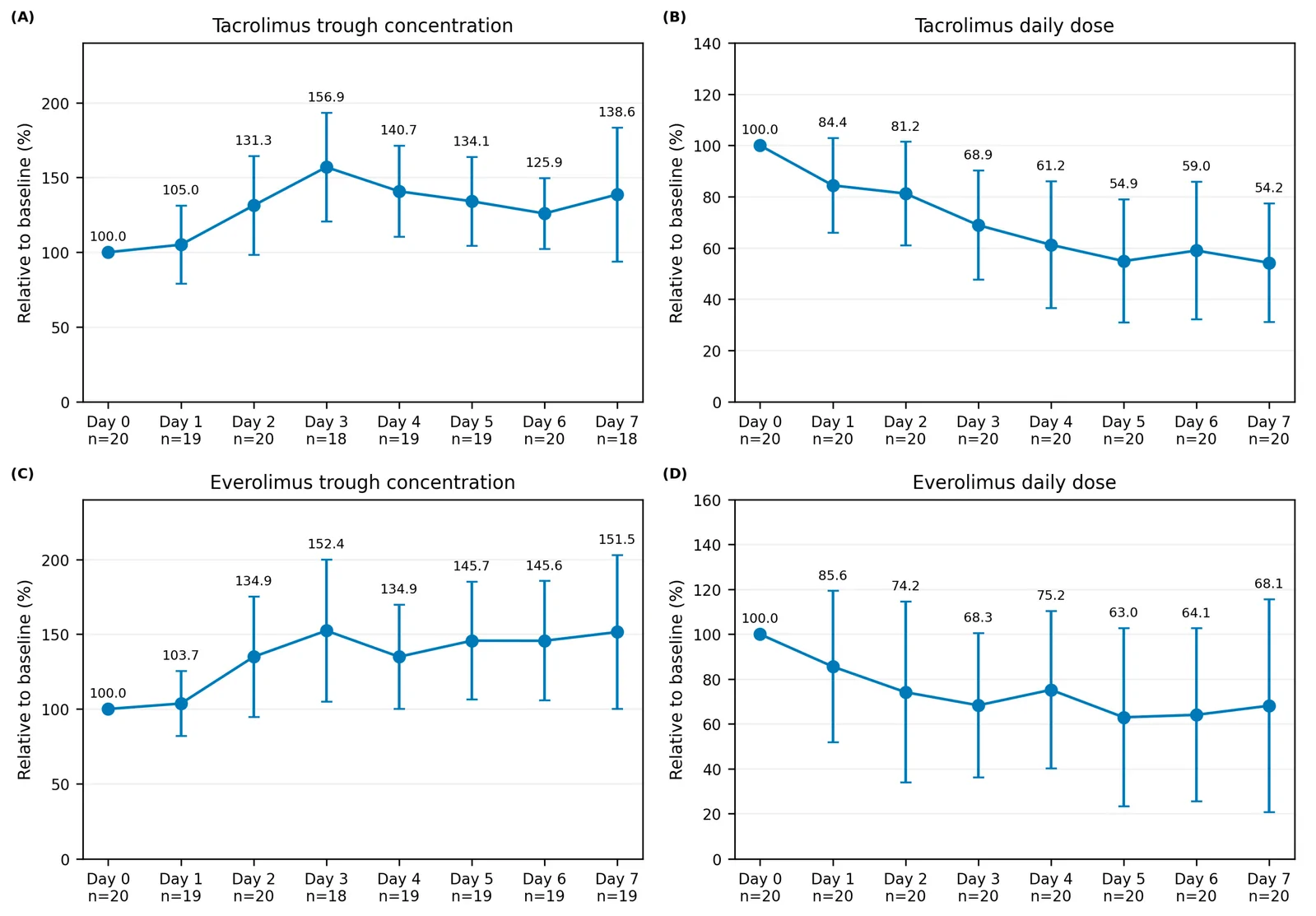
Changes in tacrolimus and everolimus trough concentrations and daily doses after letermovir initiation. (**A**) Tacrolimus trough concentration, (**B**) tacrolimus daily dose, (**C**) everolimus trough concentration, and (**D**) everolimus daily dose, expressed as percentages of the individual day 0 value for days 0–7 after letermovir initiation. Data are presented as mean ± sample SD. Mean percentages are labeled above each point, and n at each time point is shown below the x-axis. Missing measurements were not imputed.

**Figure 2:**
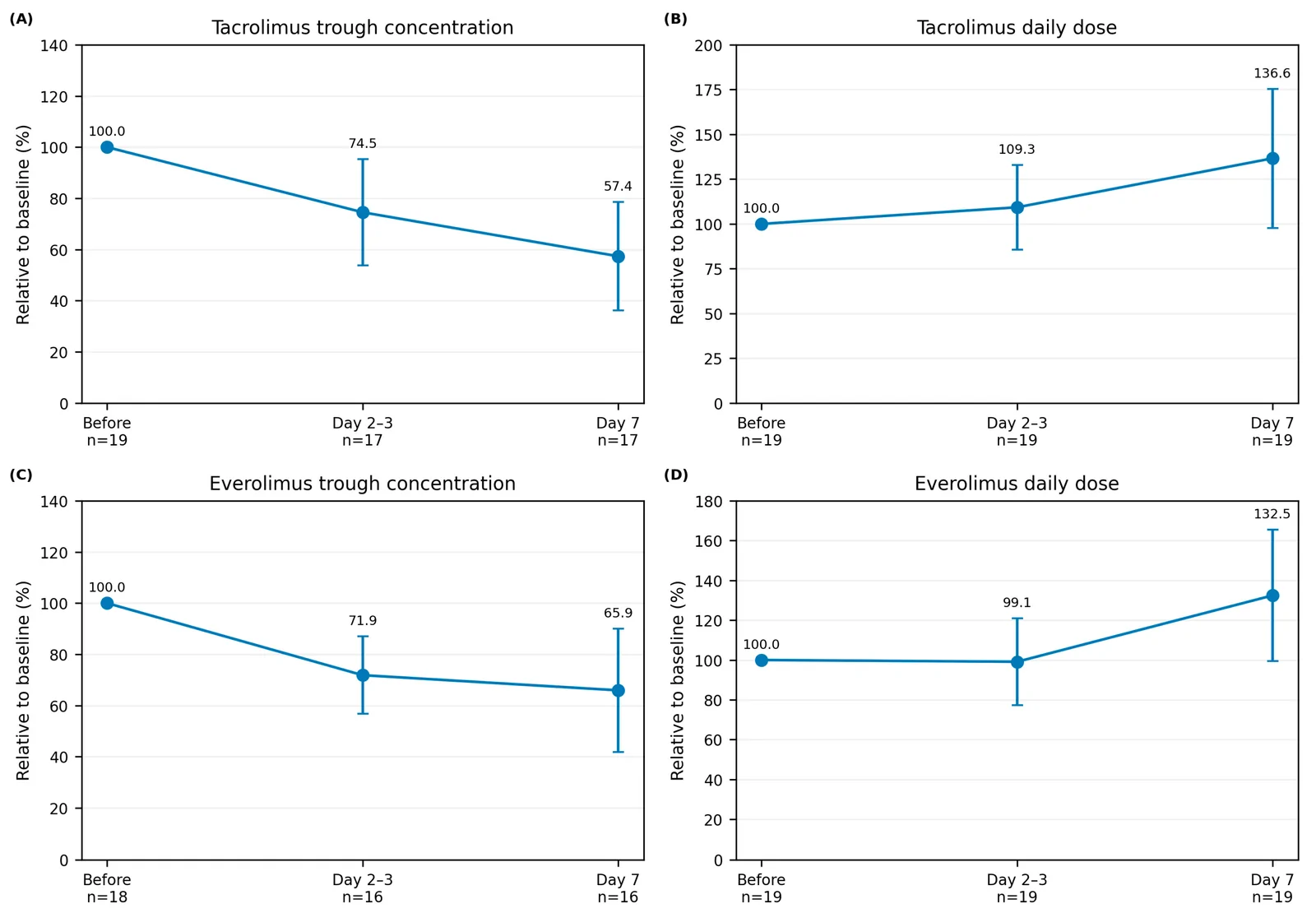
Changes in tacrolimus and everolimus trough concentrations and daily doses after letermovir discontinuation. (**A**) Tacrolimus trough concentration, (**B**) tacrolimus daily dose, (**C**) everolimus trough concentration, and (**D**) everolimus daily dose, expressed as percentages of the individual pre-discontinuation value before discontinuation, on days 2–3, and on day 7. Data are presented as mean ± sample SD. Mean percentages are labeled above each point, and n at each time point is shown below the x-axis. Missing measurements were not imputed.

**Figure 3:**
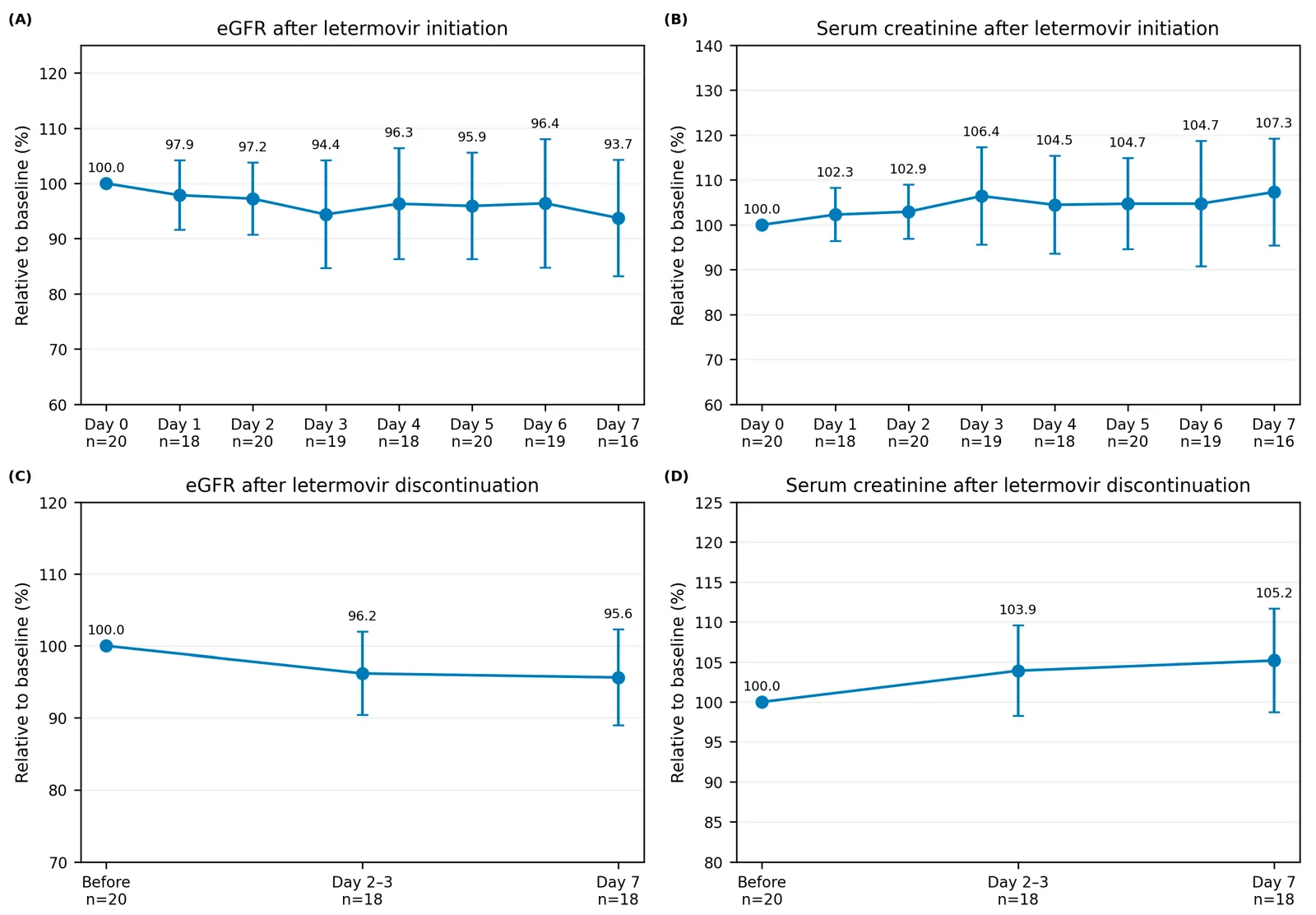
Changes in kidney allograft function after letermovir initiation and discontinuation. (**A**) eGFR and (**B**) serum creatinine, expressed as percentages of the individual day 0 value for days 0–7 after letermovir initiation; (**C**) eGFR and (**D**) serum creatinine, expressed as percentages of the individual pre-discontinuation value before discontinuation, on days 2–3, and on day 7 after letermovir discontinuation. Data are presented as mean ± sample SD. Mean percentages are labeled above each point, and n at each time point is shown below the x-axis. Missing measurements were not imputed.

For each recipient, changes in tacrolimus and everolimus trough concentrations and daily doses were expressed as percentages relative to the corresponding day 0 value. Changes in serum creatinine and eGFR were evaluated using the same approach. Missing measurements were not imputed; available observations were used at each time point, and the number of observations contributing to each longitudinal estimate is reported in Figure 1, Figure 2 and Figure 3. Each percentage change was calculated at the patient level using that recipient’s own valid baseline value; observations without a valid individual baseline or without the corresponding follow-up measurement were excluded from that time point. The reported longitudinal estimates therefore represent means of patient-level ratios rather than ratios of group means. Missing trough measurements reflected the absence of an available measurement at the prespecified clinical time point and were not treated as zero. As an exploratory analysis, dose-normalized trough concentrations (C0/D) were calculated as the measured trough concentration divided by the corresponding total daily dose when both values were available and the daily dose was greater than zero. C0/D values were also expressed relative to each recipient’s baseline C0/D. Because immunosuppressant doses were actively adjusted by therapeutic drug monitoring and steady state was not assured, C0/D was interpreted as a surrogate index of apparent exposure per unit dose rather than as a direct measure of AUC or clearance.

CMV surveillance was performed approximately monthly using quantitative CMV polymerase chain reaction (PCR). Quantitative CMV DNA testing was performed at an external reference laboratory (SRL, Inc., Tokyo, Japan) using a real-time PCR assay on the cobas 8800 System with the cobas CMV assay (Roche Molecular Systems, Inc., Branchburg, NJ, USA). CMV infection was defined as detectable CMV DNAemia exceeding 100 IU/mL. At our institution, anti-CMV therapy was initiated when CMV DNA levels reached ≥ 1000 IU/mL, irrespective of whether the patient belonged to the D+/R− or R+ group.

### 2.4. Study Endpoints

The primary endpoint was the change in tacrolimus and everolimus trough concentrations following initiation and discontinuation of letermovir. Corresponding changes in the daily doses of tacrolimus and everolimus required to maintain target trough concentrations were also evaluated.

Secondary endpoints included changes in kidney allograft function, assessed by serum creatinine and eGFR; the occurrence of CMV DNAemia, CMV syndrome, and organ-invasive CMV disease; and the safety and tolerability of letermovir prophylaxis. Safety outcomes included leukopenia, neutropenia, gastrointestinal symptoms, acute cellular rejection, antibody-mediated rejection, temporary interruption of letermovir, permanent discontinuation of letermovir, and adverse drug reactions resulting in treatment discontinuation.

Routine renal allograft biopsies were performed at the time of transplantation and approximately 1 and 6 months after transplantation, whereas episode biopsies were performed in cases of graft dysfunction. All biopsy specimens were examined by light microscopy and evaluated for C4d deposition in the peritubular capillaries by immunofluorescence staining. Acute T cell-mediated rejection and antibody-mediated rejection were diagnosed histologically based on graft biopsy findings according to the Banff 2019 criteria [12]. Borderline changes were not considered acute T cell-mediated rejection.

### 2.5. Statistical Analysis

Given the exploratory nature of this study and the limited sample size, all analyses were descriptive, and no formal hypothesis testing was performed. No formal sample size calculation was performed; all consecutive recipients who met the eligibility criteria during the study period were included. Continuous variables describing baseline characteristics were summarized as medians with interquartile ranges (IQRs), whereas categorical variables were expressed as numbers and percentages, as appropriate.

For longitudinal assessments of tacrolimus and everolimus trough concentrations, immunosuppressant doses, serum creatinine, and eGFR, data are presented as means with sample standard deviations (SDs) and as percentage changes from the respective baseline values. Day 0 was defined as the value immediately before initiation or discontinuation of letermovir, depending on the analysis. Missing data were not imputed, and analyses were based on available observations at each time point. No inferential statistical comparisons or adjustments for multiple testing were performed. In addition to the available-case analyses, a complete-case sensitivity analysis was performed for baseline-to-day-7 comparisons, restricted to recipients with valid measurements at both time points. These analyses were descriptive and were used to assess whether varying longitudinal denominators materially affected the direction or magnitude of the observed changes.

### 2.6. Ethical Considerations

This study was conducted in accordance with the Declaration of Helsinki and was approved by the Institutional Review Board of Osaka Metropolitan University on 24 August 2022 (approval protocol code 2022-086). The approved protocol included kidney transplant recipients who underwent or would undergo kidney transplantation at Osaka Metropolitan University Hospital between January 1986 and March 2032 and permitted retrospective analyses of routinely collected clinical data, including medication history, treatment course, and immunosuppressant blood concentrations. The requirement for study-specific written informed consent was waived by the Institutional Review Board because of the retrospective observational design of the study. An opt-out procedure approved by the Institutional Review Board was implemented, providing eligible patients with the opportunity to decline the use of their routinely collected clinical data for research purposes.

### 2.7. Use of Generative Artificial Intelligence in Manuscript Preparation

During preparation of this manuscript, the authors used OpenAI ChatGPT (GPT-5.6 Sol; accessed 5 September 2026) for manuscript drafting, English-language editing, structural organization, reference-format checking, verification of descriptive calculations, and preparation of figures based on author-provided data. The authors are responsible for independently reviewing and verifying all calculations, figures, scientific content, references, interpretations, and conclusions before submission. No generative artificial intelligence tool was used to generate primary study data.

## 3. Results

### 3.1. Patient Characteristics and Letermovir Exposure

Baseline recipient, donor, and transplant characteristics are summarized in Table 1. Baseline variables reported in Table 1 were complete. The median recipient age at transplantation was 52.50 years (IQR, 44.00–56.25 years); 10 recipients were male and 10 were female. The median donor age was 56.50 years (IQR, 49.00–64.00 years); 8 donors were male and 12 were female. Donor–recipient relationships included parent–child in 7 cases, siblings in 4, and spouses in 9. Sixteen recipients underwent ABO-compatible transplantation and 4 underwent ABO-incompatible transplantation. Rituximab was administered in 4 recipients. Regarding CMV serostatus, four recipients were classified as high-risk D+/R− and 16 as R+. The median interval from transplantation to letermovir discontinuation was 195.00 days (IQR, 192.00–199.25 days), and the median duration of letermovir administration was 181.00 days (IQR, 174.00–185.25 days).

### 3.2. Changes in Tacrolimus and Everolimus Trough Concentrations and Doses After Letermovir Initiation

During the 7-day period following initiation of letermovir, no concomitant medications known to affect tacrolimus or everolimus trough concentrations were initiated, discontinued, or dose-adjusted. Mean tacrolimus trough concentrations increased rapidly, reaching 156.9% of baseline on day 3 and 138.6% on day 7, while the mean daily tacrolimus dose decreased to 54.2% of baseline by day 7 (Figure 1A,B). Mean everolimus trough concentrations showed a similar increase, reaching 152.4% on day 3 and 151.5% on day 7; the mean daily everolimus dose decreased to 68.1% of baseline by day 7 (Figure 1C,D). These changes were apparent within the first several days and were managed by close therapeutic drug monitoring and individualized dose adjustment. In the exploratory dose-normalized analysis, mean tacrolimus C0/D increased to 303.3% of baseline by day 7 (SD, 179.5%; *n* = 18), and mean everolimus C0/D increased to 281.3% of baseline (SD, 138.3%; *n* = 19), consistent with increased trough exposure per unit daily dose during letermovir coadministration.

### 3.3. Changes After Letermovir Discontinuation

During the 7-day period following letermovir discontinuation, no concomitant medications known to affect tacrolimus or everolimus trough concentrations were initiated, discontinued, or dose-adjusted. Mean tacrolimus trough concentrations decreased to 74.5% of baseline on days 2–3 (*n* = 17) and 57.4% on day 7 (*n* = 17) (Figure 2A). In response, the mean daily tacrolimus dose increased to 109.3% and 136.6% of the pre-discontinuation dose on days 2–3 and day 7, respectively (*n* = 19 at each time point; Figure 2B). Mean everolimus trough concentrations decreased to 71.9% of baseline on days 2–3 and 65.9% on day 7 (*n* = 16 at each time point; Figure 2C). The corresponding mean everolimus dose remained near baseline on days 2–3 (99.1%) and increased to 132.5% by day 7 (*n* = 19 at each time point; Figure 2D). The recipient converted from tacrolimus to cyclosporine A did not contribute to the tacrolimus discontinuation analysis. In the exploratory dose-normalized analysis, mean tacrolimus C0/D decreased to 70.3% of baseline on days 2–3 (SD, 22.4%; *n* = 17) and to 44.2% on day 7 (SD, 18.5%; *n* = 17). For everolimus C0/D, the cyclosporine-converted recipient was excluded because concomitant cyclosporine required a lower letermovir dose and could confound interpretation; mean everolimus C0/D decreased to 73.0% of baseline on days 2–3 (SD, 18.8%; *n* = 15) and to 47.9% on day 7 (SD, 18.9%; *n* = 15). A sensitivity analysis excluding this recipient from the primary everolimus post-discontinuation assessment yielded results similar to the main analysis: the day-7 everolimus trough concentration and daily dose were 62.0% (*n* = 15) and 131.5% (*n* = 18) of baseline, respectively.

### 3.4. Kidney Allograft Function

Group-level kidney allograft function showed only modest changes during both initiation and discontinuation of letermovir (Figure 3), although individual variability was observed. During the 7 days after initiation, mean eGFR and serum creatinine remained within approximately 10% of baseline; at day 7, mean eGFR was 93.7% of baseline (*n* = 16) and mean serum creatinine was 107.3% (*n* = 16). Following letermovir discontinuation, mean eGFR was 96.2% on days 2–3 and 95.6% on day 7 (*n* = 18 at both time points), while mean serum creatinine was 103.9% and 105.2%, respectively (*n* = 18 at both time points). No clinically apparent deterioration in allograft function accompanied the observed group-level changes in tacrolimus and everolimus trough concentrations. Complete-case baseline-to-day-7 analyses showed the same direction and similar magnitude of change as the available-case analyses, supporting the robustness of the group-level findings despite varying longitudinal denominators.

### 3.5. CMV Outcomes and Safety

No CMV DNAemia, CMV syndrome, or organ-invasive CMV disease was observed during the analyzed period of letermovir prophylaxis among either the four high-risk D+/R− recipients or the 16 intermediate-risk R+ recipients (Table 2). Because the median letermovir discontinuation occurred at approximately post-transplant day 195, the CMV observation period predominantly reflected outcomes during active prophylaxis and did not provide a systematic assessment of post-prophylaxis CMV events.

Letermovir prophylaxis was generally well tolerated. No cases of leukopenia, neutropenia, acute T cell-mediated rejection, or antibody-mediated rejection were observed. Gastrointestinal symptoms occurred in three recipients. One recipient required temporary interruption of letermovir because of diarrhea and dehydration. After tacrolimus was changed to cyclosporine A, the diarrhea improved and letermovir was subsequently resumed. No recipient permanently discontinued letermovir because of an adverse event or adverse drug reaction (Table 3).

## 4. Discussion

In this single-center retrospective observational study, we characterized real-world changes in tacrolimus and everolimus trough concentrations and dose requirements associated with both initiation and discontinuation of letermovir in de novo kidney transplant recipients receiving an everolimus-based reduced-tacrolimus regimen. The principal findings were that letermovir initiation was followed by rapid increases in trough concentrations of both immunosuppressants requiring substantial dose reductions; discontinuation produced the reverse pattern, requiring dose escalation; kidney allograft function remained clinically stable during these changes; and no CMV DNAemia or CMV disease was observed during the analyzed period of letermovir prophylaxis. The study therefore extends existing drug-interaction evidence by providing serial clinical observations in de novo kidney transplantation, including the early post-discontinuation period.

The changes in tacrolimus trough concentrations are pharmacologically plausible and consistent with previous pharmacokinetic and clinical observations. Letermovir inhibits CYP3A, a major pathway involved in tacrolimus metabolism [6]. In a phase 1 study in healthy participants, McCrea et al. showed that letermovir increased tacrolimus AUC approximately 2.4-fold and maximum concentration approximately 1.6-fold, and recommended frequent concentration monitoring during letermovir administration and at discontinuation [7]. Importantly, the 2.4-fold value refers to AUC rather than trough concentration. In a clinical solid-organ transplant cohort, Winstead et al. observed a clinically important tacrolimus interaction after conversion to letermovir and recommended an empirical tacrolimus dose reduction of approximately 40–50% at initiation [13]. The approximately 45% tacrolimus dose reduction required in our cohort is consistent with that clinical experience.

Our findings also provide clinically relevant information regarding concomitant everolimus. Direct clinical pharmacokinetic studies of the letermovir–everolimus interaction remain limited. Menzel et al. used physiologically based pharmacokinetic modeling to predict that letermovir would increase everolimus AUC by approximately 2.5-fold, supporting therapeutic drug monitoring during coadministration [8]. This 2.5-fold estimate is model-based rather than a measured clinical AUC ratio. In a 2025 multicenter real-world kidney-transplant series, only four recipients received everolimus; among three who continued it, trough concentrations increased and two required 50% dose reductions [9]. In our cohort, everolimus trough concentrations increased by approximately 40% after letermovir initiation, necessitating a 35–40% dose reduction. These data add serial clinical trough information in a more uniform de novo transplant setting.

The reciprocal changes after letermovir discontinuation are clinically important. Tacrolimus and everolimus trough concentrations fell substantially within the first week after withdrawal, and dose requirements subsequently increased. Prior interaction guidance emphasizes concentration monitoring not only during letermovir administration but also when letermovir is stopped [6,7]. Our findings reinforce this point and suggest that the immediate post-discontinuation period is another vulnerable window for underexposure to maintenance immunosuppression. Because the magnitude and timing of dose recovery differed between tacrolimus and everolimus, individualized adjustment based on serial trough measurements appears preferable to applying the same fixed empirical dose change to both agents. The exploratory C0/D analysis showed concordant changes in exposure per unit dose, with marked increases during letermovir coadministration and reciprocal decreases after withdrawal. These findings strengthen the pharmacologic plausibility of the interaction but should not be interpreted as direct estimates of clearance or AUC because dose adjustments occurred contemporaneously and measurements were obtained under routine clinical conditions rather than at pharmacokinetic steady state. Importantly, the tacrolimus target range applicable at the pre-discontinuation baseline remained unchanged during the 7-day post-discontinuation window, and there was no scheduled protocol-driven dose reduction or target change during that interval. Thus, the observed dose escalation after letermovir withdrawal is unlikely to be explained by the routine 6-month target transition alone, although residual confounding inherent to the retrospective design cannot be excluded.

Despite the changes in trough concentrations, group mean serum creatinine and eGFR remained within approximately 10% of baseline, and no acute T cell-mediated or antibody-mediated rejection was observed. The broader feasibility of everolimus with reduced calcineurin inhibitor exposure is supported by the randomized TRANSFORM trial, in which everolimus plus reduced-exposure calcineurin inhibitor therapy was noninferior to mycophenolic acid plus standard-exposure calcineurin inhibitor therapy for the primary composite efficacy/renal-function endpoint [14]. Nevertheless, our study was small and descriptive; group-level stability in 20 recipients cannot establish renal safety or exclude clinically meaningful toxicity in individual recipients during transient immunosuppressant overexposure. The appropriate interpretation is that the observed fluctuations were manageable in this cohort when intensive therapeutic drug monitoring and prompt dose adjustment were used.

The absence of CMV DNAemia or disease during letermovir prophylaxis is noteworthy but should not be interpreted as evidence that the immunosuppressive regimen itself provided additional protection against CMV. In the randomized phase 3 trial of high-risk D+/R− kidney transplant recipients, letermovir was noninferior to valganciclovir for the prevention of CMV disease through week 52 and was associated with substantially less leukopenia or neutropenia [2]. A Japanese phase 3 study including both D+/R− and R+ kidney transplant recipients likewise demonstrated effective CMV prevention during letermovir prophylaxis with a favorable safety profile [15].

In the present study, letermovir prophylaxis extended through approximately post-transplant day 200 and therefore covered essentially the entire period during which CMV outcomes were evaluated. Consequently, the absence of CMV DNAemia or disease is consistent with the expected effect of letermovir prophylaxis and cannot be attributed independently to everolimus. Previous kidney transplant studies have reported lower rates of CMV infection with everolimus-based immunosuppression than with mycophenolate-based regimens [4,5,14,16,17]. However, those observations were obtained in different clinical settings and do not allow an independent CMV-protective effect of everolimus to be inferred in the present cohort. Because all recipients in our study received both everolimus-based immunosuppression and letermovir prophylaxis, and no comparator group was available, the respective contributions of the two treatments to CMV prevention cannot be separated.

Accordingly, the CMV findings in this study should be regarded as descriptive safety and clinical outcome data during letermovir prophylaxis rather than as evidence of additive or synergistic CMV prevention by everolimus and letermovir. Longer post-prophylaxis follow-up and comparative studies would be required to determine whether everolimus-based immunosuppression modifies the incidence of late-onset CMV after letermovir discontinuation.

Gastrointestinal symptoms were observed in three recipients, including one patient who temporarily discontinued letermovir because of diarrhea and dehydration. Although gastrointestinal adverse events have been reported during letermovir prophylaxis, controlled studies have not demonstrated a clear letermovir-specific gastrointestinal toxicity signal. In a phase 3 kidney transplant trial, the incidences of diarrhea and nausea were comparable between letermovir and valganciclovir recipients [2]. Similarly, gastrointestinal disorders occurred at comparable frequencies in letermovir- and placebo-treated hematopoietic cell transplant recipients [18], and a recent randomized trial reported identical rates of diarrhea in the letermovir and placebo groups [19]. These findings suggest that gastrointestinal symptoms during letermovir prophylaxis may be nonspecific and influenced by the post-transplant setting or concomitant medications.

In our patient with diarrhea and dehydration, symptoms improved after tacrolimus was replaced with cyclosporine A, and letermovir was subsequently resumed. This clinical course argues against letermovir as the sole cause and suggests a possible contribution from tacrolimus or its pharmacokinetic interaction with letermovir. However, causality remains uncertain because of the small sample size and concomitant medication use.

Several limitations should be acknowledged. First, this was a retrospective single-center study with only 20 recipients and no comparator group; the analyses were descriptive and cannot establish causality or comparative efficacy. The retrospective design may also have introduced selection bias and residual confounding. Although consecutive eligible recipients were included and concomitant medication changes known to affect tacrolimus or everolimus exposure were reviewed during the principal assessment periods, unmeasured confounding cannot be excluded. Second, drug exposure was assessed using trough concentrations rather than full pharmacokinetic measures such as AUC; this study should therefore be interpreted as a real-world therapeutic drug monitoring study rather than a formal pharmacokinetic investigation. Third, only four recipients were in the highest-risk D+/R− CMV category. Fourth, the study provides limited information about delayed-onset CMV after prophylaxis; current international guidance recognizes the importance of risk-adapted post-prophylaxis surveillance in selected recipients [1]. Fifth, because all recipients received both everolimus-based immunosuppression and letermovir prophylaxis, and because letermovir was administered throughout essentially the entire period of CMV assessment, the present study cannot determine whether everolimus provided any independent or additional protection against CMV. The absence of a mycophenolate-based or letermovir-free comparator group further precludes separation of the respective effects of the two treatments. Sixth, missing longitudinal measurements resulted in varying sample sizes across time points; available-case analysis may introduce bias if missingness was not random. Seventh, dose-normalized trough concentration (C0/D) was an exploratory surrogate measure obtained during active therapeutic drug monitoring and non-steady-state conditions; it cannot be equated with formal pharmacokinetic estimates of AUC or clearance. Varying denominators resulted primarily from unavailable routine clinical measurements at specific time points and, for tacrolimus after discontinuation, from conversion of one recipient to cyclosporine A. Complete-case sensitivity analyses were directionally consistent with the primary findings, but bias from nonrandom missingness cannot be excluded. Finally, the generalizability of our findings may be limited because this study was conducted at a single Japanese center and included only living-donor kidney transplant recipients receiving everolimus-based reduced-tacrolimus immunosuppression. Further studies are required to determine whether these findings are applicable to deceased-donor transplantation, other immunosuppressive regimens, and more diverse patient populations. In addition, because letermovir was continued until approximately post-transplant day 200 and the CMV analysis predominantly covered the prophylaxis period, this study provides little systematic information regarding delayed or post-prophylaxis CMV infection.

## 5. Conclusions

In de novo kidney transplant recipients receiving everolimus-based reduced-tacrolimus immunosuppression, letermovir initiation and discontinuation were associated with rapid and reversible changes in tacrolimus and everolimus trough concentrations and dose requirements. Intensive therapeutic drug monitoring at both the start and withdrawal of letermovir enabled individualized dose adjustment without clinically apparent group-level deterioration in kidney allograft function in this small cohort. These findings provide practical real-world information for management of this three-drug combination but do not establish prophylactic efficacy or a fixed dosing algorithm. Larger prospective studies are needed to define evidence-based immunosuppressant dose-adjustment strategies and to characterize CMV outcomes after discontinuation of letermovir, particularly in recipients receiving different maintenance immunosuppressive regimens.

## Figures and Tables

**Table 1:** Recipient, donor, and transplant characteristics.

Variable	Median [IQR]
Age at transplantation (years)	52.50 [44.00–56.25]
Recipient gender (male/female)	10/10
Donor age (years)	56.50 [49.00–64.00]
Donor gender (male/female)	8/12
Relationship between donor and recipient (parent–child/sibling/spouse)	7/4/9
ABO compatibility (compatible/incompatible)	16/4
Rituximab administration (+/−)	4/16
CMV high risk/intermediate risk	4/16
Duration from transplantation to letermovir discontinuation (days)	195.00 [192.00–199.25]
Letermovir administration duration (days)	181.00 [174.00–185.25]

Abbreviations: IQR, interquartile range; CMV, cytomegalovirus. Values are presented as median [interquartile range] or number of patients.

**Table 2:** CMV outcomes during letermovir prophylaxis through approximately post-transplant day 200.

Outcome	High-Risk CMV Group (*n* = 4)	Intermediate-Risk CMV Group (*n* = 16)
CMV infection (DNAemia)	0	0
CMV disease (CMV syndrome)	0	0
Organ-invasive CMV disease	0	0

Abbreviation: CMV, cytomegalovirus.

**Table 3:** Safety and adverse events within 6 months after transplantation.

Variable	*n*
Leukopenia (<3000/µL)	0
Neutropenia (<1500/µL)	0
Acute T cell-mediated rejection	0
Antibody-mediated rejection	0
Gastrointestinal symptoms	3
Adverse events requiring temporary interruption of letermovir *	1
Adverse events leading to permanent discontinuation of letermovir	0
Adverse drug reactions leading to permanent discontinuation	0

* One recipient required temporary interruption because of diarrhea and dehydration. Diarrhea improved after switching tacrolimus to cyclosporine A, and letermovir was subsequently resumed.

## Data Availability

The data presented in this study are available from the corresponding author on reasonable request, subject to institutional and ethical restrictions. The data are not publicly available because they contain information that could compromise participant privacy.

## Abbreviations

The following abbreviations are used in this manuscript:

Abbreviation	Definition
ABMR	antibody-mediated rejection
AUC	area under the concentration–time curve
CMV	cytomegalovirus
CNI	calcineurin inhibitor
C0/D	dose-normalized trough concentration
D+/R−	donor CMV-seropositive/recipient CMV-seronegative
eGFR	estimated glomerular filtration rate
EVR	everolimus
IQR	interquartile range
MPA	mycophenolic acid
PCR	polymerase chain reaction
PBPK	physiologically based pharmacokinetic
SD	standard deviation
TAC	tacrolimus
TDM	therapeutic drug monitoring

## References

[1] Kotton C.N., Kumar D., Manuel O., Chou S., Hayden R.T., Danziger-Isakov L., Asberg A., Tedesco-Silva H., Humar A., Transplantation Society International CMV Consensus Group (2025). The Fourth International Consensus Guidelines on the Management of Cytomegalovirus in Solid Organ Transplantation. Transplantation.

[2] Limaye A.P., Budde K., Humar A., Vincenti F., Kuypers D.R.J., Carroll R.P., Stauffer N., Murata Y., Strizki J.M., Teal V.L. (2023). Letermovir vs. Valganciclovir for Prophylaxis of Cytomegalovirus in High-Risk Kidney Transplant Recipients: A Randomized Clinical Trial. JAMA.

[3] Budde K., Kamar N., Crespo M., Small C.B., Stauffer N., Broyde N., Gilbert C.L., Moise P., Haber B. (2025). Impact of Kidney Function on 200 Days of Antiviral Prophylaxis for Cytomegalovirus Disease in Cytomegalovirus-Seronegative Recipients of Cytomegalovirus-Seropositive Donor Kidneys: Post Hoc Analysis of a Randomized, Phase 3 Trial of Letermovir vs. Valganciclovir Prophylaxis. Am. J. Transplant..

[4] Tedesco-Silva H., Felipe C., Ferreira A., Cristelli M., Oliveira N., Sandes-Freitas T., Aguiar W., Campos E., Gerbase-DeLima M., Franco M. (2015). Reduced Incidence of Cytomegalovirus Infection in Kidney Transplant Recipients Receiving Everolimus and Reduced Tacrolimus Doses. Am. J. Transplant..

[5] Kaminski H., Kamar N., Thaunat O., Bouvier N., Caillard S., Garrigue I., Anglicheau D., Rérolle J.-P., Le Meur Y., Durrbach A. (2022). Incidence of Cytomegalovirus Infection in Seropositive Kidney Transplant Recipients Treated with Everolimus: A Randomized, Open-Label, Multicenter Phase 4 Trial. Am. J. Transplant..

[6] Burger D.M., Nijboer L., Ghobreyal M., Maertens J., Blijlevens N., Hilbrands L., Baas M.C., Ljungman P., Brüggemann R.J.M. (2024). Drug–Drug Interaction Management with the Novel Anti-Cytomegalovirus Agents Letermovir and Maribavir: Guidance for Clinicians. Clin. Pharmacokinet..

[7] McCrea J.B., Macha S., Adedoyin A., Marshall W., Menzel K., Cho C.R., Liu F., Zhao T., Levine V., Kraft W.K. (2019). Pharmacokinetic Drug–Drug Interactions between Letermovir and the Immunosuppressants Cyclosporine, Tacrolimus, Sirolimus, and Mycophenolate Mofetil. J. Clin. Pharmacol..

[8] Menzel K., Kuo Y., Chen D., Hartmann G., Wang Y.-H., Cho C.R., McCrea J.B., Asari K. (2023). Developing a Mechanistic Understanding of the Nonlinear Pharmacokinetics of Letermovir and Prospective Drug Interaction with Everolimus Using Physiologically Based Pharmacokinetic Modeling. Clin. Transl. Sci..

[9] Benotmane I., Schvartz B., Garrouste C., Runyo F., Boud’hors C., Flahaut G., Danthu C., Bertrand D., Caillard S. (2025). Real-World Evaluation of Letermovir Use in Kidney Transplant Recipients: Drug Interactions, Safety, and Impact on Renal Function. Transpl. Int..

[10] von Elm E., Altman D.G., Egger M., Pocock S.J., Gøtzsche P.C., Vandenbroucke J.P., STROBE Initiative (2007). The Strengthening the Reporting of Observational Studies in Epidemiology (STROBE) statement: Guidelines for reporting observational studies. Lancet.

[11] Matsuo S., Imai E., Horio M., Yasuda Y., Tomita K., Nitta K., Yamagata K., Tomino Y., Yokoyama H., Hishida A. (2009). Revised equations for estimated GFR from serum creatinine in Japan. Am. J. Kidney Dis..

[12] Loupy A., Haas M., Roufosse C., Naesens M., Adam B., Afrouzian M., Akalin E., Alachkar N., Bagnasco S., Becker J.U. (2020). The Banff 2019 Kidney Meeting Report (I): Updates on and Clarification of Criteria for T Cell- and Antibody-Mediated Rejection. Am. J. Transplant..

[13] Winstead R.J., Kumar D., Brown A., Yakubu I., Song C., Thacker L., Gupta G. (2021). Letermovir Prophylaxis in Solid Organ Transplant—Assessing CMV Breakthrough and Tacrolimus Drug Interaction. Transpl. Infect. Dis..

[14] Pascual J., Berger S.P., Witzke O., Tedesco H., Mulgaonkar S., Qazi Y., Chadban S., Oppenheimer F., Sommerer C., Oberbauer R. (2018). Everolimus with Reduced Calcineurin Inhibitor Exposure in Renal Transplantation. J. Am. Soc. Nephrol..

[15] Ishida H., Goto N., Imamura R., Sasaki H., Unagami K., Futamura K., Murata Y., Oshima N., Eto T., Haber B. (2024). Letermovir Safety and Efficacy for Cytomegalovirus Prophylaxis in Adult Japanese Kidney Transplant Recipients: A Multicenter, Open-Label, Noncomparative Phase 3 Study. Clin. Exp. Nephrol..

[16] Berger S.P., Sommerer C., Witzke O., Tedesco H., Chadban S., Mulgaonkar S., Qazi Y., de Fijter J.W., Oppenheimer F., Cruzado J.M. (2019). Two-year outcomes in de novo renal transplant recipients receiving everolimus-facilitated calcineurin inhibitor reduction regimen from the TRANSFORM study. Am. J. Transplant..

[17] Devresse A., Leruez-Ville M., Scemla A., Avettand-Fenoel V., Morin L., Lebreton X., Tinel C., Amrouche L., Lamhaut L., Timsit M.O. (2018). Reduction in late onset cytomegalovirus primary disease after discontinuation of antiviral prophylaxis in kidney transplant recipients treated with de novo everolimus. Transpl. Infect. Dis..

[18] Chemaly R.F., Ullmann A.J., Stoelben S., Richard M.P., Bornhäuser M., Groth C., Einsele H., Silverman M., Mullane K.M., Brown J. (2014). Letermovir for cytomegalovirus prophylaxis in hematopoietic-cell transplantation. N. Engl. J. Med..

[19] Russo D., Schmitt M., Pilorge S., Stelljes M., Kawakita T., Teal V.L., Haber B., Bopp C., Dadwal S.S., Badshah C. (2024). Efficacy and safety of extended duration letermovir prophylaxis in recipients of haematopoietic stem-cell transplantation at risk of cytomegalovirus infection: A multicentre, randomised, double-blind, placebo-controlled, phase 3 trial. Lancet Haematol..

